# Infantile atopic dermatitis and maternal-infant bonding: a mixed methods study

**DOI:** 10.1186/s13223-023-00857-5

**Published:** 2023-11-29

**Authors:** Ayel Luis R. Batac, Kaitlyn A. Merrill, Michael A. Golding, Manvir Bhamra, Zoe Harbottle, Isac Kopsch, Erik Wilking, Marina Jonsson, Sandra Ekström, Elissa M. Abrams, Michelle A. Halbrich, Elinor Simons, Leslie E. Roos, Jill A. Keddy-Grant, Thomas V. Gerstner, Jo-Anne St-Vincent, Jennifer L. P. Protudjer

**Affiliations:** 1 Department of Pediatrics and Child Health, Max Rady College of Medicine, Rady Faculty of Health Sciences, University of Manitoba, Winnipeg, MB Canada; 2 Children’s Hospital Research Institute of Manitoba, Winnipeg, MB Canada; 3 Karolinska Institutet, Stockholm, Sweden; 4 Institute of Environmental Medicine, Karolinska Institutet, Stockholm, Sweden; 5 Centre for Occupational and Environmental Medicine, Stockholm County Council, Stockholm, Sweden; 6 Department of Clinical Science and Education, Södersjukhuset, Karolinska Institutet, Stockholm, Sweden; 7 Department of Pediatrics and Child Health, Section of Allergy and Clinical Immunology, Max Rady College of Medicine, Rady Faculty of Health Sciences, University of Manitoba, Winnipeg, MB Canada; 8 Division of Allergy and Immunology, Department of Pediatrics, Faculty of Medicine, University of British Columbia, Vancouver, BC Canada; 9 Children’s Allergy and Asthma Education Centre, Health Sciences Centre Winnipeg, Winnipeg, MB Canada; 10 Department of Psychology, Faculty of Arts, University of Manitoba, Winnipeg, MB Canada; 11 Department of Pediatrics and Child Health, Section of Dermatology, Max Rady College of Medicine, Rady Faculty of Health Sciences, University of Manitoba, Winnipeg, MB Canada; 12 Department of Food and Human Nutritional Sciences, Faculty of Agricultural and Food Sciences, University of Manitoba, Winnipeg, MB Canada; 13 George and Fay Yee Centre for Healthcare Innovation, Winnipeg, MB Canada

**Keywords:** Atopic dermatitis, Maternal-infant bonding, Maternal health, Maternal mental health, Postpartum bonding questionnaire, Mixed methods

## Abstract

**Background:**

Childhood atopic dermatitis can have a negative effect on caregivers’ quality of life and stress levels due to the burdensome nature of its treatment. Given that the condition often emerges in infancy, atopic dermatitis-related stress also carries the potential to negatively affect the developing mother-infant bond. While it is plausible that atopic dermatitis has a negative impact on maternal-infant bonding, these relationships have not been studied directly. In light of this gap, the current study investigated the association between infantile atopic dermatitis and the maternal-infant bond using a mixed-method design.

**Methods:**

Mothers of infants (< 19 months) with atopic dermatitis were recruited from social media and medical clinics between October 2021 and May 2022. Mothers with infants unaffected by inflammatory skin conditions were also recruited to serve as a control group. Participants were asked to complete questionnaires related to their demographics, child’s health, and mother-infant bond. Multiple linear regression analyses were used to assess bonding quality among cases and controls. A subset of cases were also asked to participate in semi-structured interviews focused on infantile atopic dermatitis and the maternal-infant bond.

**Results:**

The final sample consisted of 32 cases and 65 controls. Scores on the impaired bonding and risk of abuse subscales did not significantly differ between cases and controls. However, mothers of infants with atopic dermatitis did report lower levels of caregiving anxiety (*b* = − 1.47, *p* < 0.01) and pathological anger/rejection (*b* = − 1.74, *p* = 0.02) relative to controls. Qualitative findings suggest that the topical therapies required to manage atopic dermatitis may strengthen the bond between some mothers and infants.

**Conclusion:**

Findings suggest that atopic dermatitis does not have a negative impact on maternal-infant bonding and may actually improve bonds in some cases. In light of this finding, clinicians may leverage the potentially positive impact of atopic dermatitis-related caregiving on the maternal-infant bond to encourage caregivers to remain adherent to their child’s topical treatments.

**Supplementary Information:**

The online version contains supplementary material available at 10.1186/s13223-023-00857-5.

## Background

Atopic dermatitis (AD), also known as eczema, is a chronic, often relapsing condition, that causes patches of dry, itchy skin [1]. Although the condition can present at any age, AD typically presents before the age of 1 year [1]. Epidemiological research has found that AD is common among children in developed countries, with a recent systematic review and meta-analysis finding a pooled of 12-month prevalence 8.3–16.3%, depending on the child’s age [2].

Although AD is chronic in nature, treatments are available to reduce the frequency of flares and minimize their severity and duration when they do occur. This is typically predicated on avoiding triggers and maintaining the skin’s moisture through regular bathing and the application of emollients [3]. Symptom exacerbations, on the other hand, are often treated through the use of topical corticosteroids [3]. For patients with moderate-severe AD, wet-wrap therapy may also be recommended to be used in concert with emollients and topical medications (e.g., corticosteroids, calcineurin inhibitors) during periods of acute flares [3]. Although treatments for AD are typically topical, phototherapy and/or systemic immunomodulatory agents may be used in cases were AD does not respond to first-line treatments [4].

While topical therapies have been found to be effective in relieving symptoms and preventing flares, research suggests that their time-consuming nature can impose a significant burden on caregivers [5, 6]. In fact, a study by Su and colleagues suggests parents devote 2–3 h daily to caring for their child with AD, depending on the severity of the condition [5]. Because of its burdensome nature, caregivers also tend to report lower quality of life and higher levels of stress relative to caregivers of children without AD [6–8].

Given that AD often emerges in the first year of life, mothers are likely to be saddled with this additional stress during a critical period for the mother-infant relationship [1]. During this time, mothers are typically in the process of developing a close emotional connection or bond with their infant, in a process that has been compared to “falling in love” with their baby [9]. For most mothers, this connection is manifested in positive feelings, emotional warmth, and physical affection, which in turn promotes the socioemotional development of the infant [9, 10]. Some mothers, however, fail to develop this close emotional bond, which may have negative consequences on the mother-infant relationship and the socioemotional development of the child [10].

Research suggests that maternal mental health plays an important role in the formation of healthy mother-infant bonds [11]. To date, much of this research has focused on postpartum depression, but some evidence has linked greater stress and anxiety to lower quality bonds [11–14]. In light of this finding, it stands to reason that the burden of caring for a child with AD may have a deleterious impact on the emotional bond between a mother and her infant. Consistent with this reasoning, research has found less positive patterns of interaction (e.g., less warmth, sensitivity, etc.) between the mothers of infants with other health conditions and vulnerabilities, including preterm infants and those with congenital heart defects (CHD), over the first six months of life [15, 16]. It should be noted, however, that the findings tend to be more mixed when bonding is assess through parent-report, with some studies providing evidence of impaired bonding, some returning null findings, and others finding a positive impact [15–18].

While it is plausible that AD has a negative impact on maternal-infant bonding, it is important to note that these relationships have not been studied directly. In light of this gap, we utilized a mixed-methods design to investigate the relationship between AD and mother-infant bonding. By incorporating both quantitative and qualitative measures, we hoped to not only understand whether differences in bonding are found between mothers with an infant with AD and those without, but also how AD affects this relationship.

## Methods

### Study design

The current study employed a mixed-methods, sequential explanatory design. First, quantitative data were derived from a series of self-report questionnaires. Second, a subset of participants who had a child with AD were asked to complete qualitative interviews aimed at achieving a deeper understanding of their bonding experiences when caring for a child with AD. Findings from the qualitative interviews were then used to help contextualize and explain the quantitative results [19].

### Participant recruitment

Mothers who had an infant under the age of 19 months with AD were recruited from both social media and allergy/dermatology clinics in Winnipeg, Manitoba, Canada between October 2021 and May 2022. Using the same means, we also recruited mothers with infants of the same age who had not been diagnosed with AD or any other uncomfortable skin condition to serve as a comparison group. After providing their informed consent, participants were asked to complete a series of online questionnaires that assessed their household’s demographics, allergic disease history, mental health history, and maternal-infant bonding.

### Quantitative measures

#### Sociodemographics and allergic disease history

Participants were asked to complete an ad hoc questionnaire containing items related to age, gender, race/ethnicity, education, relationship status, income, household composition, mental health history, allergic disease history, and recent stressful events. Mothers of infants with AD were asked a number of additional questions related to how they manage their child’s condition.

#### Maternal–infant bonding

Maternal–infant bonding was assessed via the Postpartum Bonding Questionnaire (PBQ), a 25-item questionnaire designed to assess the quality of a mother’s emotional attachment to their infant [20]. Each item is scored on a 6-point Likert scale with higher scores indicating more impaired bonding. Items are scored to form four subscales: impaired bonding, pathological rejection/anger, anxiety about caregiving, and risk of abuse. Cut scores have also been developed to help identify mothers with potentially problematic bonds. In particular, scores above 11, 12, 9, and 1 on the impaired bonding, rejection/anger, anxiety about caregiving, and risk of abuse scales are considered indicative of impaired bonding, respectively [21].

#### Atopic dermatitis severity

Mothers with a child with AD were asked to rate the severity of their child’s condition using the Patient-Oriented SCORing Atopic Dermatitis index (i.e., PO-SCORAD), a self-report questionnaire and that allows patients or caregivers to rate the severity of their own or their child’s AD [22]. In completing the measure, respondents are provided with a series of pictures depicting symptoms characteristic of AD at varying levels of severity. Respondents are then asked to select the picture for each symptom that best matches their own or their child’s skin. Respondents are also asked to rate itchiness and sleep quality on a ten-point, visual analog scale. Based on their responses, respondents are assigned a score ranging from 0–103 with higher scores indicating more severe AD. According to work by Silverberg and colleagues, individuals scoring less than 28 are best described as having mild AD, scores between 28–56 are considered moderate, and scores greater than 57 are characteristic of severe AD. [23]

### Quantitative analyses

Prior to the substantive analyses, the dataset was assessed for non-valid responding and missing data. This analysis revealed several variables had missing data, ranging in amount from 1–7%. In order to maintain power and limit the potential for bias, multiple imputation was used to create 50 datasets. Following imputation, a series of multiple linear regression analyses were used to assess differences in maternal-infant bonding between mothers with an infant with AD and those without. Multiple linear regression was also used to examine the relationship between AD severity and maternal-infant bonding. Each analysis was adjusted for the infant’s food allergy status (i.e., yes vs. no), number of adults in the household as well as the mother’s age, relationship status, employment status, history of allergic disease, education, whether target child is the mother’s first, and whether the mother had other children with AD. Covariates were selected using the modified disjunctive cause criterion outlined by VanderWeele [24]. Statistical significance was set at α = 0.05. All analyses were conducted using Stata 17.0 (StataCorp, College Station, TX, USA).

### Qualitative data collection

A subset of mothers who had an infant with AD also completed semi-structured qualitative interviews focused on better understanding the relationship between AD and the maternal infant bond. All mothers approached to participate in the interviews had consented during the quantitative portion of the study to be contacted about future research related to the overarching bonding and AD project. Mothers who consented to the qualitative arm were asked to participate in a phone interview in which they were asked to discuss the burden of caring for a child with AD as well as if they perceived their child’s AD as affecting their mental health, sleep, and their bond (Please see Additional file 1 for the list of questions included in the interview guide). Each interview was recorded and transcribed verbatim by a trusted, third-party transcription service, before being analyzed.

### Qualitative analysis

**Fig. 1:**
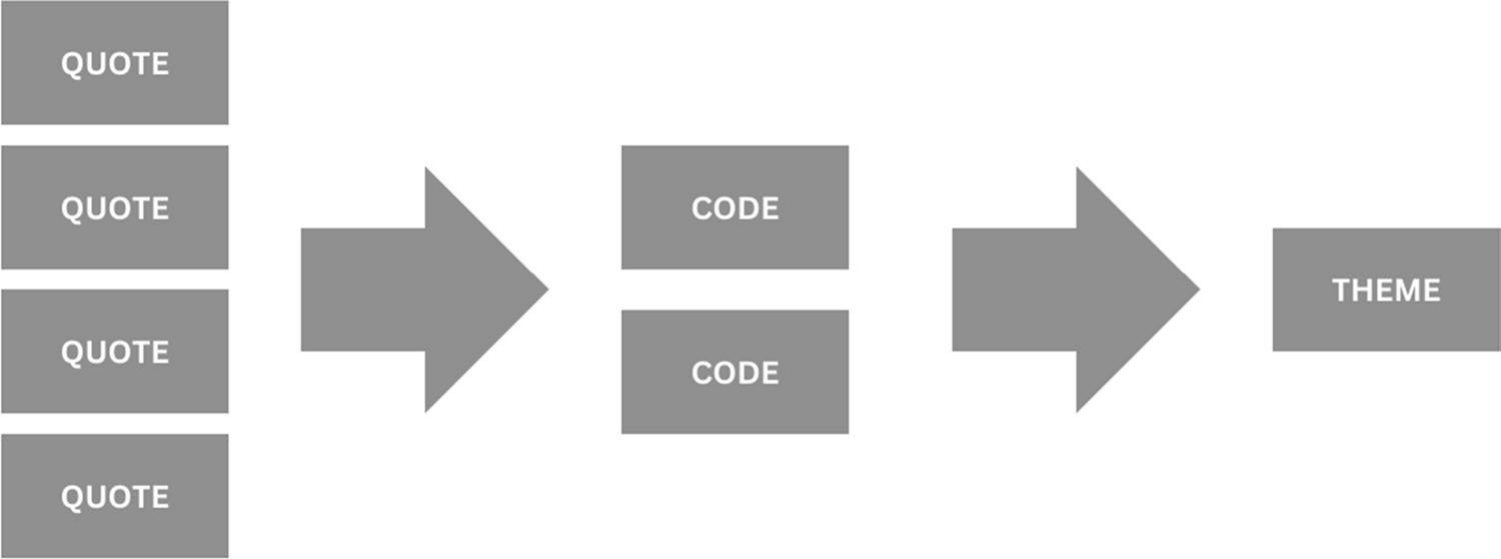
Flow chart demonstrating the process of thematic analysis

Transcripts from the interviews were analyzed using thematic analysis (see Fig. 1). Thematic analysis is a qualitative analysis technique used to identify and analyze patterns or themes within qualitative data [25]. Using this technique, the analyst organizes the data by applying short descriptions, referred to as codes, to relevant sections of text. Once the relevant sections are coded, the codes are organized into overarching themes, which describe broader patterns in the data related to the research question. In the present study, the transcripts were analyzed by one coder and subsequently reviewed by a second analyst to ensure there was consensus on how the data was interpreted.

## Results

### Quantitative results

#### Participant characteristics

The final sample included 32 cases and 65 controls. Participants were about 31 years old on average (full sample: *M* = 30.9, *SD* = 4.2; cases: *M* = 31.3, *SD* = 4.2; controls: *M* = 30.1, *SD* = 4.2) and had approximately two children (full sample: *M* = 1.8, *SD* = 0.9; cases: *M* = 1.6, *SD* = 0.8; controls *M* = 1.9, *SD* = 1.0). Most participants were White (61%), with smaller numbers of Indigenous (18%) and Asian (11%) participants represented. At the time of the study, most participants indicated they were on “some type of leave (e.g., maternity, disability, sick leave, etc.)” (63%) from their job. Smaller numbers of participants reported being employed full-time (14%), part-time (7%), or unemployed (12%). Participating children had an average age of about 8 months (full sample: *M* = 7.6, *SD* = 4.3; cases: *M* = 8.2, *SD* = 4.6; controls: *M* = 7.2, *SD* = 4.1) and included roughly equal numbers of males and females in the case and control groups (cases: 40% females; controls: 51% females; Please see Table 1 for a full summary of the demographic findings). A series of independent samples *t*-tests and *χ*^*2*^ tests revealed cases and controls did not significantly differ on any of the demographic variables apart from employment status. As such, we controlled for the participant’s work status in each of the substantive analyses.

**Table 1:** Demographic and clinical characteristics

	Cases			Controls		
	%	n	Mean (*SD*)	%	n	Mean (*SD*)
Mother’s age		32	30.1 years (4.2)		65	31.3 years (4.2)
Child’s age		32	8.2 months (4.6)		63	7.2 months (4.1)
Mother’s relationship status						
In a relationship	90	28		92	59	
Single	10	3		8	5	
Child’s sex						
Female	41	13		51	33	
Male	59	19		49	32	
Mother’s education						
< High school diploma	19	6		15	10	
Some post-secondary training	12	4		9	6	
Post-secondary degree/ diploma	69	22		75	49	
Household income						
< $51,000	32	10		24	14	
$51,000-$99,000	36	11		37	22	
≥ $100,000	32	10		39	23	
Maternal allergic comorbidities						
Atopic dermatitis	47	14		22	14	
Food allergy	12	4		2	1	
Asthma	28	9		29	19	
Rhinitis	16	5		3	2	
Child allergic comorbidities						
Food allergy	25	8		8	5	

Cases largely rated their child’s AD as mild to moderate in severity as only one participant fell in the severe range (PO-SCORAD > 57; *M* = 25.4, SD = 13.8). On the PBQ, mothers with a child with AD were found to have mean scores of 5.8 (*SD* = 3.95), 2.3 (*SD* = 1.8), 2.2 (*SD* = 2.4), and 0 (*SD* = 0) on the impaired bonding, anxiety with caregiving, pathological rejection/anger, and risk of abuse scales, respectively. By comparison, mothers in the control group reported higher scores on each of these scales (impaired bonding: *M* = 7.1, *SD* = 4.4; anxiety with caregiving: *M* = 3.5, *SD* = 2.3; rejection/anger: *M* = 3.6, *SD* = 3.3; risk of abuse: *M* = 0.03, *SD* = 0.2). Across both groups, the majority of mothers had no scores on any of the four PBQ subscales that exceeded the cut-offs indicative of disordered bonding (cases = 78%; controls = 86%).

#### Associations between atopic dermatitis and bonding

Results from a series of multiple linear regression analyses revealed significant differences between cases and controls on two of the four PBQ subscales. Relative to controls, mothers with a child with AD reported significantly lower scores on the scales related to anxiety about caregiving (*b* = − 1.47, 95% CI = − 2.49, − 0.45, *p* < 0.01) and pathological rejection/anger (*b* = − 1.74, 95% CI = − 3.17, − 0.31, *p* = 0.02). Cases did not, however, significantly differ from controls on the subscales related to impaired bonding or risk of abuse (Please see Table 2 for a full summary). While mothers with a child with AD reported higher quality bonds on some subscales, the severity of a child’s AD was not found to significantly predict bonding quality on any of the PBQ subscales (all *p* > 0.05).

**Table 2:** Adjusted multiple regression analyses predicting maternal-infant bonding quality from infantile AD status

	*b*	SE	95% CI	*p*-value
Impaired bonding				
Child AD status	− 1.48	1.02	− 3.50; 0.55	0.15
Pathological rejection/anger				
Child AD status	− 1.74	0.72	− 3.17; − 0.31	0.02
Caregiving anxiety				
Child AD status	− 1.47	0.51	− 2.49; − 0.45	0.005
Risk of abuse				
Child AD status	− 0.04	0.05	− 0.14; 0.05	0.38

Cases were coded as 1 and controls as 0 on the child AD status variable. The infant’s food allergy status (i.e., yes vs. no), number of adults in the home as well as the mother’s age, relationship status, employment status, allergic disease history, education, whether the mother had a child/children previously, and whether the mother had other children with AD were included as covariates in each of the analyses

*AD* atopic dermatitis, *95% CI* 95th percent confidence interval

### Qualitative results

The final qualitative sample included ten mothers with infants with AD. Most participants were in their late 20 s and early 30 s (*M* = 29.2, *SD* = 5.0) and had one child in the home (*M* = 1.4, SD = 0.5). Similar to the quantitative findings, 60% of participants were White, 20% were Indigenous, and 10% were Asian, with the remainder reporting their race as “other”. The vast majority of participants were married (90%) at the time of the study and reported living with just one other adult (70%). At the time of the interview, most participants were on “leave” from their job (70%), with smaller numbers working full-time or part-time (20%). Participants tended to have a post-secondary education (70%) and half reported an annual household income of $100,000 Canadian dollars or more. Their infants with AD were mostly male (80%) and ranged in age from 1 to 18 months (*M* = 8.5 months, *SD* = 4.9). The majority of these children had PO-SCORAD scores consistent with mild (30%) to moderate AD (60%).

Through the qualitative interviews, one overarching theme related to maternal-infant bonding was identified: AD can have a positive influence on bonding.

Contrary to expectations, mothers did not describe AD as having a negative impact on their bond. Surprisingly, participants either indicated AD had no impact or they emphasized the perceived positive impacts on bonding. Rather than describing the additional caregiving needs of their infant as solely burdensome, many noted how they provided opportunities for nurturance that would not have been available otherwise. In particular, some mothers described how the need to regularly bath and moisturize their baby promoted a greater degree of physical and emotional closeness: “I think eczema makes us closer in some ways, making sure that I am putting his cream on, and we always have our daily massages with his lotion and stuff like that, just to make sure that he is being taken care of.”—Participant 1, male child, 1 month old.

Emotional closeness was not only predicated on physical touch, however, as participants also described how the discomfort caused by their infant’s AD evoked feelings of tenderness and an empathic desire to alleviate their child’s suffering. For some mothers, the alleviation of their infant’s suffering brought with it a satisfying sense of purpose as their infant was dependent on them for relief: “I am taking care of him more, it is a better relationship because I always have to look after him and I always have to think about him, in a good way. ‘Cause I know that he needs me, he needs me to take care of him more.”—Participant 3, male child, 7 months.

While a number of mothers emphasized the positive contributions of AD on bonding, other participants explained that their child’s condition had little impact on their emotional or physical connection: “I would say that we have a really good relationship, regardless of the eczema.”—Participant 4, female child, 8 months. Interestingly, none of the mothers described AD as having a negative impact on their relationship with their baby. That being said, one mother did indicate that she was hesitant to caress her infant’s face out of fear of irritating his inflamed skin. However, this limitation was not perceived as having a negative effect on their bond (See Table 3 for a table of qualitative codes and representative quotations).

**Table 3:** Primary theme, qualitative codes and supportive quotations

Theme	Codes	Quotes
AD can benefit the maternal-infant bond	AD elicits empathy	“It’s not like ‘oh gross, I don’t want to touch it’. Its more ‘that looks uncomfortable. I want to fix it’.” -Participant 1, male child, 1 month “it was really hard to see him so uncomfortable and so bothered by it” -Participant 7, male child, 18 months
	Increased caregiving	“I think it [AD] makes us closer in some ways. I’m making sure that I am putting his cream on, and like we always have our daily massages with his lotion…just to make sure that he is being taken care of” -Participant 1, male child, 1 month “I am taking care of him more, like it is a better relationship because I always have to look after him and I always have to think about him, in a good way. Cause I know that he needs me, he needs me to take care of him more” -Participant 3, male child, 7 months
	Few negative impacts on the mother-infant relationship	“I would say that we have a really good relationship, regardless of the eczema” -Participant 4, female child, 8 months Interviewer: “Do you think your relationship with your youngest has been affected by his eczema in any way?” Participant:—“I don't think so. I think that we just have the relationship we have with him because of, you know, who he is and his little personality” -Participant 8, male child, 14 months

## Discussion

The current study was the first, to our knowledge, to investigate the relationships between AD and maternal-infant bonding. Contrary to expectations, findings did not support the hypothesized relationships between AD status and the mother-infant bond. On the contrary, mothers with a child with AD reported higher quality bonds, relative to controls, on the subscales related to caregiving anxiety and pathological rejection/anger. These findings were, in part, corroborated by the qualitative data as mothers reported that they perceived their child’s AD as having a neutral or positive influence on their bond.

Through the interviews, a number of mothers noted that the additional caregiving that their child’s AD required, rather than creating resentment, helped to forge closer bonds by promoting physical intimacy and emotional tenderness. In particular, mothers described how the discomfort experienced by their infant evoked a desire to alleviate their child’s suffering, which helped to foster emotional closeness. Moreover, the need for frequent moisturization was also described positively as it provided opportunities for bonding through physical touch. In light of these comments, it is not surprising that mothers reported significantly lower levels of pathological rejection/anger as this subscale contains items related to both emotional and physical closeness.

In addition to reporting lower levels of pathological rejection/anger, cases in the current study also reported experiencing less caregiving anxiety relative to controls. Unfortunately, the qualitative interviews provided little insight into how AD affected mothers’ confidence in caring for their baby; however, evidence from the psychological literature suggests that the lower levels of caregiving anxiety reported by mothers with a child with AD may stem from a process of exposure and adjustment [26, 27]. Because infantile AD requires additional caregiving over and above the typical demands of raising an infant, it stands to reason that it may help reduce caregiving anxiety by increasing a mother’s confidence through increased exposure to caregiving. Consistent with this reasoning, research in other populations has shown that caregiving anxiety tends to decrease with increased caregiving experience [26, 27].

The current findings linking pediatric AD to higher quality maternal-infant bonds, while surprising, are somewhat consistent with more recent research showing higher quality bonds among the mothers of preterm infants [17, 18]. While it is not known for certain, it has been hypothesized that the higher quality bonds reported by some mothers of premature infants may stem from their more intensive caregiving practices. Beckwith and Cohen argued that caregivers with medically fragile infants engage in more caregiving in a bid to compensate for their child’s negative experiences, and in the case of pre-mature infants, to provide them with stimulation aimed at addressing their potential developmental delays [28]. While the additional caregiving provided by the parents of children with AD is simply aimed at treating their skin condition, it is interesting to note that in both cases, these more intensive caregiving practices have been linked to higher quality patterns of interaction or bonds.

That being said, some studies have provided evidence that pediatric medical conditions and vulnerabilities may in fact have a negative impact on the mother-infant relationship [15, 16, 29, 30]. For instance, a Korean study by Im and colleagues found mothers with a child with AD reported showing less affection, a potential corollary of bonding, relative to mothers who did not have a child with AD [29]. In light of these contradictory findings, it is apparent that more research is needed to better understand the factors that moderate the relationship between pediatric health conditions and mother-infant bonding.

Nevertheless, findings from the current study highlighting the benefits of AD-related caregiving on bonding provide early evidence that, if reliable, may be used to promote greater treatment adherence among the caregivers of children with AD. Research suggests that adherence to topical treatments for AD is poor, due in part, to their burdensome and time consuming nature [31]. However, caregivers may be more willing to persist with these treatments with the awareness that they may not only benefit their infant’s skin, but also their bond with their infant.

While the current study provides novel information concerning the relationship between AD and maternal-infant bonding, it is not without limitations, including it’s relatively small sample size. While we had hoped to recruit larger numbers of both cases and controls, our recruitment efforts were unfortunately hampered by the fact that data was collected during the early waves of the COVID-19 pandemic. During this time, many medical appointments occurred virtually, which limited the number of participants available to be recruited in clinic. It should also be noted that we did not collect data pertaining to whether participants, or their infants, had a history of health conditions beyond allergic disease and mental health issues, in the case of the caregivers. Because the case and control groups were quite similar demographically, we have some reason to believe that their physical well-being was roughly comparable, but in the absence of data, we cannot know for sure. For this reason, it is possible that the bonding differences between cases and controls may have stemmed, fully or partially, from health conditions other than AD.

The current study was also limited by the fact that only one infant was classified as having severe AD. Therefore, it is difficult to determine whether the findings extend beyond milder manifestations of the condition. It is also difficult to definitively determine whether the findings generalize, or transfer in the case of the qualitative findings, across racial backgrounds  and income strata [32]. Overall, participants tended to be White or Indigenous, and in the case of the qualitative interviewees, relatively affluent. As such, it is not clear whether the findings fully generalize to individuals of other races and income levels. In light of these limitations, future research should strive to determine the degree to which the current findings are replicable in larger and more diverse samples.

## Conclusion

Findings from the current study suggest that infantile AD does not have a deleterious impact on the mother-infant bond, at least among infants with mild to moderate AD. In fact, the routine caregiving required to manage AD may enhance some mothers’ perceptions of their bond with their infant. While more research is needed to assess the reproducibility of these findings, the current study suggests that the potentially positive impact of AD-related caregiving on the mother-infant bond may be leveraged by clinicians to encourage mothers to stay adherent to their infant’s topical therapies despite their burdensome nature.

### Supplementary Information

**Additional file 1.** Qualitative Interview guide.

## Data Availability

Datasets generated and analyzed during the current study are not publicly available out of respect for participant privacy. However, the data can be made available upon reasonable request.

## Abbreviations

AD: Atopic dermatitis
CHD: Congenital heart defects
PBQ: Postpartum Bonding Questionnaire
PO-SCORAD: Patient-Oriented Scoring of Atopic Dermatitis
