# Supplementary material for: Infantile atopic dermatitis and maternal-infant bonding: a mixed methods study
Source: Allergy Asthma Clin Immunol. 2023 Nov 29;19:100. doi: 10.1186/s13223-023-00857-5 (PMC10687835; doi:10.1186/s13223-023-00857-5)
Supplement: Supplementary file 1 — Additional file 1. Qualitative Interview guide. [file 13223_2023_857_MOESM1_ESM.docx]

Additional File 1 – Qualitative interview guide

1. Could please tell me a little bit about yourself and your family?
2. How has your life changed since the birth of your baby?
3. When was your baby diagnosed with AD?
   1. Follow-up questions to establish month/season when the diagnosis was made.
   2. How severe is your baby’s eczema?
4. Please describe the effort that it takes to care for your child’s atopic dermatitis.
   1. Follow-up question: How has caring for your infant with AD impacted your levels of stress or mood?
5. Has AD affected your baby’s mood or behaviour? If so, please tell me how.
6. Please describe both your baby’s and your sleeping patterns.
7. Follow up question: How has your baby’s AD impacted your sleep?
8. Has your relationship with your baby been affected by AD?
   1. Follow-up question: Has it impacted physical closeness? If yes, how so?
   2. Follow-up question: Has it affected your emotional connection to your infant? If yes, how so?
9. Specific to mothers with other children without AD: How do you experience caring for your infant with AD compared to your other children?
10. What kinds of supports do you have for childcare?
    1. Follow up question: How do your baby’s treatments for AD impact on your ability to find childminders that you can trust?
    2. Follow up question: What additional supports would you like, if any?
11. What kinds of supports do you have for yourself (housework, split bills, errands)?
    1. Follow up question: What additional supports would you like, if any?
12. What concerns do you have for your child, now and in the future?
13. What advice would you give to other mothers whose infant has been just diagnosed with AD?
